# Improving Constraints on Planetary Interiors With PPs Receiver Functions

**DOI:** 10.1029/2021JE006983

**Published:** 2021-11-02

**Authors:** D. Kim, V. Lekić, J. C. E. Irving, N. Schmerr, B. Knapmeyer‐Endrun, R. Joshi, M. P. Panning, B. Tauzin, F. Karakostas, R. Maguire, Q. Huang, S. Ceylan, A. Khan, D. Giardini, M. A. Wieczorek, P. Lognonné, W. B. Banerdt

**Affiliations:** ^1^ Department of Geology University of Maryland College Park College Park MD USA; ^2^ Institute of Geophysics ETH Zürich Zürich Switzerland; ^3^ School of Earth Sciences University of Bristol Bristol UK; ^4^ Bensberg Observatory University of Cologne Cologne Germany; ^5^ Max Planck Institute for Solar System Research Göttingen Germany; ^6^ Jet Propulsion Laboratory California Institute of Technology Pasadena CA USA; ^7^ Université de Lyon UCBL ENSL CNRS LGL‐TPE Villeurbanne France; ^8^ Research School of Earth Sciences Australian National University Acton ACT Australia; ^9^ Istituto Nazionale di Geofisica e Vulcanologia, Sezione di Bologna Bologna Italy; ^10^ Department of Computational Mathematics, Science, and Engineering Michigan State University East Lansing MI USA; ^11^ Department of Physics New Mexico State University Las Cruces NM USA; ^12^ Université Côte d'Azur Observatoire de la Côte d'Azur CNRS Laboratoire Lagrange Nice France; ^13^ Université de Paris Institut de Physique du Globe de Paris CNRS Paris France

**Keywords:** *InSight*, Seismology, Receiver function, Mars, Martian crust, Transdimensional hierarchical Bayesian

## Abstract

Seismological constraints obtained from receiver function (RF) analysis provide important information about the crust and mantle structure. Here, we explore the utility of the free‐surface multiple of the P‐wave (PP) and the corresponding conversions in RF analysis. Using earthquake records, we demonstrate the efficacy of PPs‐RFs before illustrating how they become especially useful when limited data is available in typical planetary missions. Using a transdimensional hierarchical Bayesian deconvolution approach, we compute robust P‐to‐S (Ps)‐ and PPs‐RFs with *InSight* recordings of five marsquakes. Our Ps‐RF results verify the direct Ps converted phases reported by previous RF analyses with increased coherence and reveal other phases including the primary multiple reverberating within the uppermost layer of the Martian crust. Unlike the Ps‐RFs, our PPs‐RFs lack an arrival at 7.2 s lag time. Whereas Ps‐RFs on Mars could be equally well fit by a two‐ or three‐layer crust, synthetic modeling shows that the disappearance of the 7.2 s phase requires a three‐layer crust, and is highly sensitive to velocity and thickness of intra‐crustal layers. We show that a three‐layer crust is also preferred by S‐to‐P (Sp)‐RFs. While the deepest interface of the three‐layer crust represents the crust‐mantle interface beneath the *InSight* landing site, the other two interfaces at shallower depths could represent a sharp transition between either fractured and unfractured materials or thick basaltic flows and pre‐existing crustal materials. PPs‐RFs can provide complementary constraints and maximize the extraction of information about crustal structure in data‐constrained circumstances such as planetary missions.

## Key Points

- We compute robust P‐to‐S (Ps)‐, P‐waves (PPs)‐, and S‐to‐P (Sp)‐receiver functions (RFs) to infer properties of the crustal layers beneath the *InSight* lander in Elysium Planitia
- Sp‐RFs as well as observed variability of the 7.2 s phase between Ps‐ and PPs‐RFs require a three‐layer crust
- PPs‐RFs can provide complementary constraints for crustal imaging on Earth and other planetary bodies

## 1 Introduction

Planetary crusts preserve information about the thermal and magmatic history of a planet. Our understanding of the evolution of Earth's interior has been informed by the velocity and density structure of its crust. Seismology can offer direct constraints on crustal layering and properties. For example, due to the presence of strong impedance contrast across the Mohorovičić discontinuity (or Moho; Mohorovičić, 1910), seismic body waves convert from compressional to shear (*Ps*) or vice versa, and seismologists commonly use these conversions in receiver function (RF) analysis (Burdick & Langston, 1977; Langston, 1977; Phinney, 1964; Vinnik, 1977) to study variations in crustal thickness as well as internal P and S wave seismic velocity structures (e.g., Zhu & Kanamori, 2000). Some studies have employed RF analysis to investigate even deeper structures in the lithosphere (e.g., Fischer et al., 2010) or the mantle transition zone (e.g., Dueker & Sheehan, 1997; Lawrence & Shearer, 2006; Munch et al., 2020; Tauzin et al., 2008), and over the last 45 years, RF analysis has become a standard tool of body wave seismology on Earth.

Seismic recordings collected on the Moon and Mars by the *Apollo* and *InSight* mission (e.g., Banerdt et al., 2020; Lognonné et al., 2019, 2020; Nunn et al., 2020) have provided direct information on the interior structure of both planetary bodies from crust (e.g., Kovach and Watkins, 1973; Toksöz et al., 1974) to core (Garcia et al., 2011; Stähler et al., 2021; Weber et al., 2011). RF analysis of these data has led to important insights. Vinnik et al. (2001) first reported the utility of the RF analysis on the Moon by detecting the shear to compressional (S‐to‐P, Sp) conversions across the base of the uppermost regolith layer and possibly at the lunar crust‐mantle boundary. Lognonné et al. (2003) and Gagnepain‐Beyneix et al. (2006) further explored the crustal thickness of the Moon based on the RF stack from Vinnik et al. (2001) along with the reprocessed *Apollo* seismic data of 60 moonquakes. Lognonné et al. (2003) illustrated the non‐uniqueness of the RF travel times for determination of the lunar crustal thickness and the bias between low/high seismic velocities and thin/thick crust and suggested much thinner crust than that initially determined for the Moon by Toksöz et al. (1974). These thin crust models were finally confirmed by the evidence for high crustal porosity in the GRAIL lunar gravity mission (Wieczorek et al., 2013). On Mars, preliminary RFs derived from two marsquakes (Lognonné et al., 2020) showed the first evidence of subsurface layering on Mars with low seismic velocities in the first upper 8–11 km; additional observations and inversions complemented by gravitational field modeling, have enabled average crustal thickness of Mars to be constrained between 24 and 72 km, with important geochemical and geodynamical implications (Knapmeyer‐Endrun et al., 2021).

In principle, an ideal RF represents the impulse response due to structure directly beneath a seismic station, because signature of the source and source‐side structure is removed through deconvolution (e.g., Ammon, 1991). Thus, unlike many other seismic methodologies that require a network of seismometers, RF analysis can extract useful structural constraints even when a single three component seismic station is available (e.g., Burky et al., 2021; Kim and Lekić, 2019; Panning et al., 2017). Extraction of rich information contained in RFs at a single station can be achieved by incorporating many independent measurements from both *Ps*‐RFs and the corresponding free‐surface reverberations contained in the same record. For example, Kumar and Bostock (2008) demonstrate that average crustal P velocity (*V*_P_), *V*_P_/*V*_s_, and crustal thickness beneath the Global Seismic Network (GSN) station HYB in Hyderabad, India, can be extracted using both direct conversions and free‐surface multiples. Another popular approach of jointly analyzing different phases in RFs is to combine Ps‐ and Sp‐RFs. Using the same HYB station, Rychert and Harmon (2016) show how stacking Ps‐ and Sp‐RFs further enhance the sensitivity to crustal properties by eliminating the dependence on *V*_P_.

As a part of the prelanding studies of the *InSight* mission to Mars, Knapmeyer‐Endrun et al. (2018) have used synthetic seismograms generated by hypothetical marsquake events using different Mars prelanding models to show the potential availability of RFs can be extended by incorporating waveforms of free‐surface multiples of the P‐wave (PPs). Several mantle transition zone studies have incorporated PPs‐RFs in their common conversion point stacking analysis on Earth (Boyce & Cottaar, 2021; Nyblade et al., 2000; Owens et al., 2000; Shen et al., 1998) while fewer studies use them to constrain the overlying crustal structure (e.g., Jones & Phinney, 1998). The infrequent use of PPs arrivals in RF analysis for crustal studies on Earth is due to the following: (a) the signal‐to‐noise ratio (SNR) of the PPs are typically much smaller than both Ps and Sp phases, (b) signal‐generated noise that arrives within the P‐coda (e.g., pP) for a shallow event can possibly contaminate the data prior to deconvolution, (c) additional constraints obtained in comparison with solely using Ps‐RFs are relatively small due to small differences in ray parameters for distant earthquakes, but perhaps more fundamentally, and (d) earthquakes that are large enough for Ps RF analysis occur with sufficient frequency on Earth to reduce the need for additional data.

In this study, we compute Ps‐RF and explore the utility of the PPs‐RF derived from low‐frequency (LF) event recordings on Mars (e.g., J. F. Clinton et al., 2021; *InSight* Mars SEIS Data Service, 2019; *InSight* Marsquake Service, 2021). We also present Sp‐RF analysis on S0235b which has the largest SNR of all five event used in our analysis and is the only event with clear Sp signals. To produce robust RFs, we apply the transdimensional hierarchical Bayesian deconvolution (THBD) method (Kolb & Lekić, 2014) which yields an ensemble of Ps‐, PPs‐, and Sp‐RFs for each event whose features appear in proportion to their likelihood. Because this approach enables us to quantify the uncertainty associated with our RFs and to estimate parameters describing the background noise in raw data, our RFs can provide reliable structural inference at the expense of large computational cost. We first demonstrate the feasibility of our approach using a pair of earthquakes recorded by the AGMN broadband station (Albuquerque Seismological Laboratory/USGS, 1990) deployed in Agassiz National Wildlife Refuge, Minnesota whose crustal structure is relatively homogeneous with a thickness of 45–50 km (e.g., Ford et al., 2016; Shen & Ritzwoller, 2016). Next, we conduct Ps‐ and PPs‐RF analysis with the five highest‐quality LF marsquake waveforms, and discuss additional structural inference we obtain by conducting a joint analysis on ensembles of Ps‐ and PPs‐RFs.

## 2 Data and Methods

### 2.1 Earthquakes

**Figure 1:**
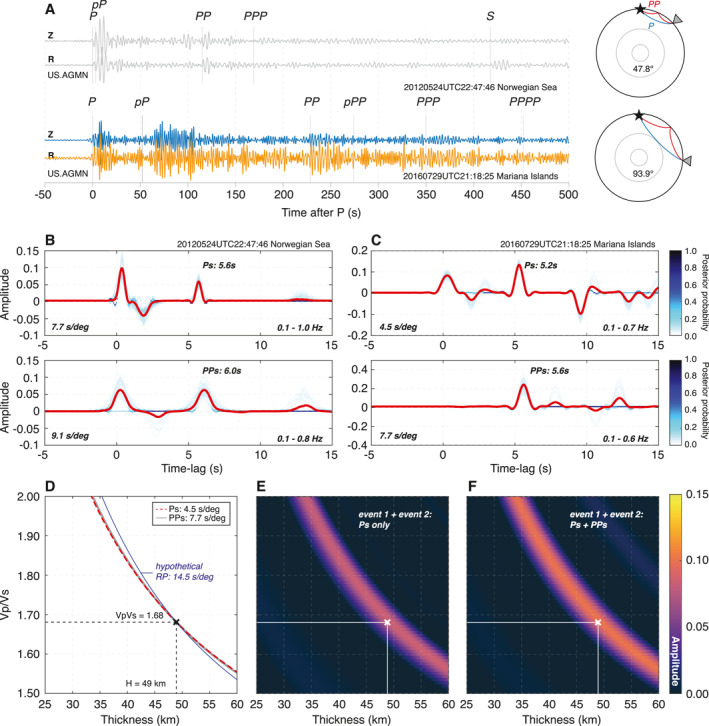
The receiver function (RF) analysis on Earth using a pair of earthquake recordings. (a) Vertical and radial component seismograms from Mw6.3 Norwegian Sea (2012‐05‐24UTC22:47:46) and Mw7.7 Mariana Islands (2016‐07‐29UTC21:18:25) earthquakes recorded by broadband station US.AGMN. Gray lines indicate body wave phases predicted by iasp91. The ray paths of P (blue) and PP (red) for each event are shown on the right. (b and c) Ensemble Ps‐ and PPs‐RFs combining all models for events in panel (a). The ensemble mean RF for each event is shown in red. Ray parameter estimates and the bandpass filter range used for processing are noted at the bottom left and right side at each panel, respectively. Note that due to the epicentral distances chosen, the Ps and PPs phases in the lower and upper panels of Figures 1B and 1C are expected to arrive at similar lag times. (d) Theoretical thickness versus *V*_P_/*V*_s_ (*H*‐*κ*) curves for Ps (dashed red) and PPs (gray) phases in panel (b) for the bulk crustal *V*_P_ of 6.5 km/s. Calculation is made for those *H*‐*κ* values estimated by EarthScope Automated Receiver Survey (black cross). The hypothetical PPs *H*‐*κ* curve for a much larger ray parameter is shown in blue. Comparison of the *H*‐*κ* stacks using average RFs in panels (b and c) with panel (e) Ps phase alone versus (f) both Ps and PPs phases. The maximum amplitude found in the *H*‐*κ* space is marked by white symbol.

We compute Ps‐ and PPs‐RF for a pair of earthquakes recorded by US.AGMN broadband station deployed at Agassiz National Wildlife Refuge, Minnesota (Figure 1). Here, the search criteria for selecting two good quality events are: (a) the presence of strong P and PP arrivals in waveform data in the 0.1–1.0 Hz frequency range; (b) excellent fit provided by the EarthScope Automated Receiver Survey (EARS; Crotwell & Owens, 2005) between data and bulk crustal properties (e.g., correlation coefficient >0.96 between the modeled and observed RFs); and (c) epicentral distance ratio of the two events of the pair should be two. The last criterion allows us to directly assess our resulting Ps‐ and PPs‐RFs because the lag time of Ps phases generated by the closer event should be in theory consistent to the lag time of PPs phases by the further event (cf., lower and upper panels of Figures 1b and 1c) given that the velocity structure remains largely the same on both ray paths (ray path diagram in Figure 1a).

We rotate the event waveforms from ZNE to ZRT components, apply a bandpass filter to each component, and estimate the upgoing P and SV energy using a free surface transform (FST; Kennett, 1991). To apply the FST to our earthquake data, we adopt the bulk crustal *V*_P_ and *V*_P_/*V*_S_ values reported by EARS, and ray parameters of the P and PP arrivals from isap91 (Kennett & Engdahl, 1991). Different bandpass filter ranges between 0.1 and 1.0 Hz are used to optimize the visibility of P and PP arrivals for each event (Figure 1a). We window the P‐component waveform to a duration of 8–10 s, starting at the P‐ or PP‐arrival to remove later arriving phases and pre‐event noise while avoiding abrupt waveform truncation.

On each pair of P and SV waveforms, we apply the transdimensional hierarchical Bayesian deconvolution (THBD, Kolb & Lekić, 2014). Unlike other popular deconvolution methods (e.g., Clayton & Wiggins, 1976; Ligorria and Ammon, 1999; Sheehan et al., 1995), this approach estimates an ensemble of RFs by performing a large number of forward simulations as each modeled RF is parameterized by Gaussian pulses with stochastically varying width, location (i.e., delay time), and amplitude. In this process, background noise is parameterized from the data covariance matrix and modeled simultaneously. An ensemble of RFs is produced for each event after five million iterations of the THBD, discarding the first half as burn‐in, and saving every 1,000th sample to the ensemble. Once convergence is achieved, RFs in the ensemble are samples of the complete posterior probability density. Hence, the resulting THBD RFs allow us to robustly quantify uncertainties on both amplitude and delay time for each converted phase at the expense of relatively high computational cost. Notably, our approach is suitable and unlikely to introduce spurious signals into the RFs, even when the SNR is low or data availability is limited (e.g., Kolb & Lekić, 2014). By using the signals around the expected timings of Ps and PPs from average Ps‐ and PPs‐RFs of the corresponding THBD RF ensembles, we present the trade‐off between crustal thickness (*H*) and the *V*_P_/*V*_S_ (*κ*) for a given bulk crustal *V*_P_ of the region (i.e., *H*‐*κ* curve).

### 2.2 Marsquakes

**Figure 2:**
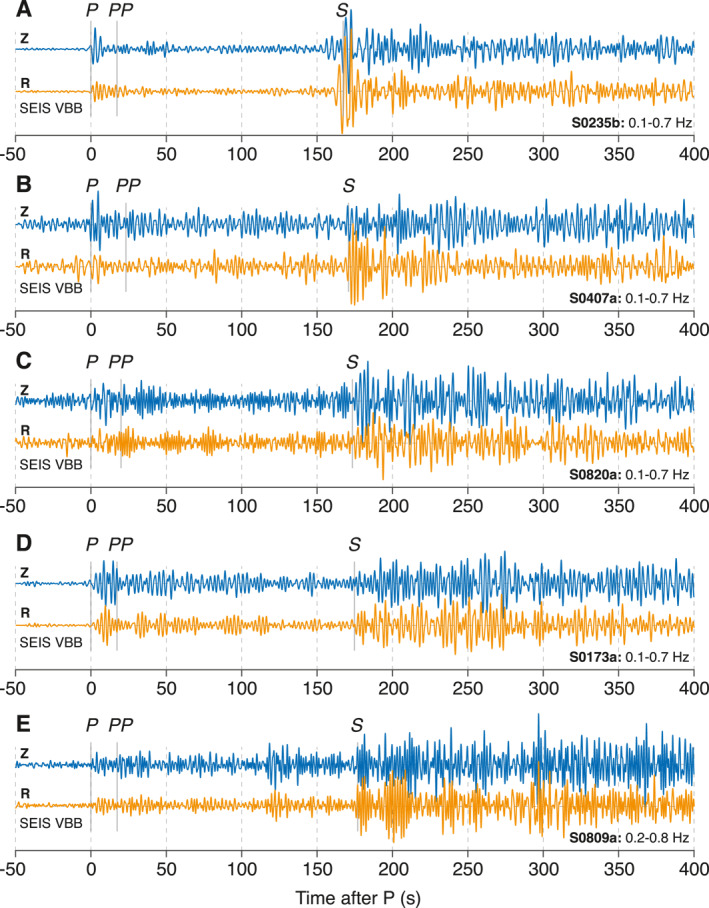
Five marsquake low‐frequency (LF) event waveforms. Vertical and radial component data from five marsquake LF events: (a) S0235b, (b) S0407a, (c) S0820a, (d) S0173a, and (e) S0809a recorded by Seismic Experiment for Interior Structure Very Broad Band. Waveforms are bandpass filtered with the frequency ranges as noted at the bottom of each panel. Phase picks are shown in gray. All five events used in this study are in The Marsquake Service event quality A‐B and show relatively high signal‐to‐noise ratio with dominant seismic energy below 1 Hz. By definition, event quality A‐B in *InSight* data denotes events that show multiple clear and identifiable phases with coherent polarization (J. F. Clinton et al., 2021; InSight Marsquake Service, 2021) allowing epicentral locations to be robustly determined (e.g., Figure 3).

**Figure 3:**
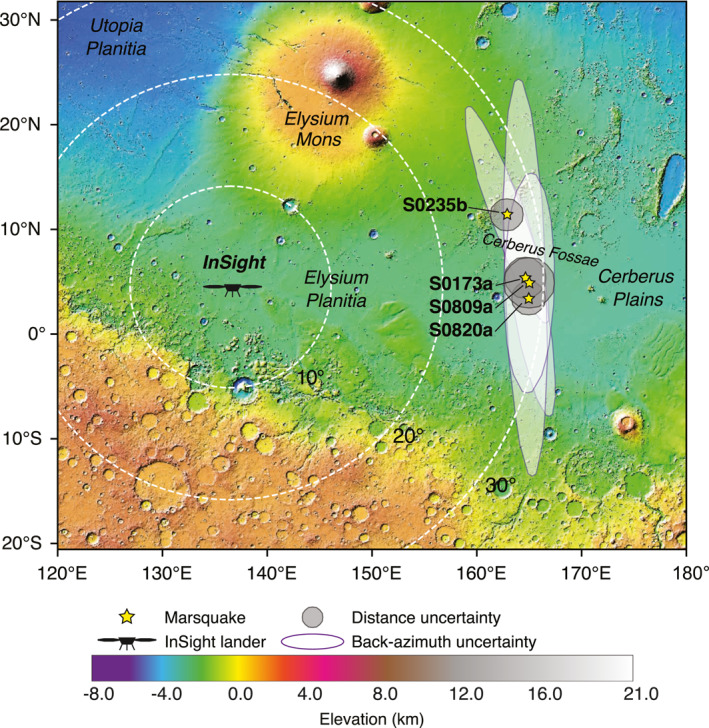
Location of marsquakes. The Marsquake Service (MQS)‐reported epicenters of marsquake events used in this study (InSight Marsquake Service, 2021) are indicated by yellow stars, while the black symbol denotes the *InSight* lander. All of these events originate in the general area of Cerberus Fossae located to the east of the *InSight* landing site at Elysium Planitia. Note S0407a is not shown due to the absence of the MQS reported back azimuth. Uncertainties associated with the event distance and back azimuth are denoted by gray circles and white ellipses, respectively. The background topography is from the Mars Orbiter Laser Altimeter (e.g., Smith et al., 1999).

We use recordings of five marsquakes–S0173a, S0235b, S0407a, S0809a, and S0820a–which have the highest SNR (e.g., The Marsquake Service [MQS; J. Clinton et al., 2018] event quality A‐B; InSight Marsquake Service, 2021) since the landing of *InSight* on Mars (Figure 2). Similar to the way, we processed earthquake data, we first rotate 20 Hz UVW channels from the Very Broad Band sensor of the Seismic Experiment for Interior Structure (SEIS; Lognonné et al., 2019) to ZNE then use back azimuths provided by the MQS and Khan et al. (2021) to rotate into the ZRT coordinates. For S0407a, a back azimuth estimate is not available from the MQS, so we estimated the back azimuth value by performing a grid‐search on a 2 s window around the P‐arrival as we maximize the ratio between the average power of radial and transverse component data. The estimated distance of these LF events is ∼30° and all originate in the general area of Cerberus Fossae, among the youngest tectonic structures on Mars (Giardini et al., 2020), located to the east of the *InSight* landing site at Elysium Planitia (Figure 3).

Seismic data acquired by SEIS on Mars include idiosyncratic signals that arise from various sensor and mechanical components of the spacecraft system. The 8 distinct classes of idiosyncratic signals that are prominent throughout the seismic data can be categorized into two types based on their duration: transient signals including glitches, spikes, and donks; and, sustained signals including lander modes, tick noise, and the 2.4 Hz resonance (see Kim et al., 2021 for detailed descriptions of these signals). We bandpass filter our data between 0.2 and Fh Hz to process Ps‐ and PPs‐RF, setting Fh = 0.7 and 0.6, respectively. This bandpass makes our data processing unlikely to suffer from those artifacts associated with 1 Hz tick noise and its overtones, lander modes, the 2.4 Hz resonance or the transient spikes and donks, which can complicate structural interpretations of data collected by the SEIS instrument (Kim et al., 2021). Known instrument glitches affecting data recordings are identified and removed, following Scholz et al. (2020). Our picks of P and PP arrival times for the LF events are guided by the vespagram approach described in Khan et al., 2021 (see Supporting Information of Khan et al., 2021) and verified against the MQS arrival times.

**Figure 4:**
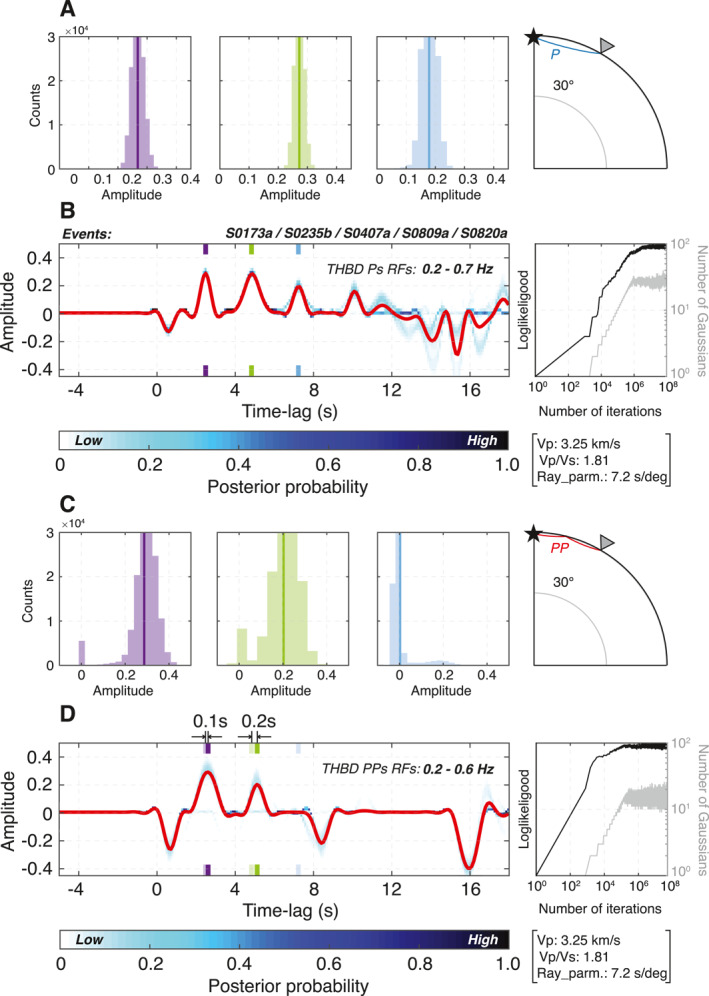
Transdimensional hierarchical Bayesian deconvolution (THBD) P‐to‐S‐ (Ps) and P‐waves (PPs)‐receiver functions (RFs) on Mars using five low‐frequency (LF) marsquake events. (a and b) Ensemble Ps‐RFs combining all models of the simultaneous analysis on five LF marsquakes that are located ∼30° away from the *InSight* lander. The ensemble mean RF is shown in red. Histograms in panel (a) indicate the corresponding relative amplitudes across the entire RF solutions in the ensemble examined at each peak (marked by purple, green, and blue ticks on panel (b)) for the first few stable phases discussed in the main text. The ray path of P (blue) is shown on the right. Our LF marsquake waveform data are bandpass filtered between 0.2 and 0.7 Hz prior to THBD. Both evolution of the model likelihood (black) and the number of Gaussian (gray) as a function of iteration are plotted on the right side of the RF ensemble. Below, we denote the average free surface transform parameters used in our analysis. Panels (c and d) same as panels (a and b), but the resulting PPs‐RF counterparts. The ray path of PP (red) is shown on the right. Color markers in panel (b) are faded in panel (d). Waveform data are bandpass filtered between 0.2 and 0.6 Hz. Note the 2.4 and 4.8 s phases in panels (a and b) are slightly delayed by 0.1 and 0.2 s, respectively in panels (c and d), while the instability of the 7.2 s is observed across the two ensembles.

To obtain the upgoing P and SV wavefields from the ZRT waveforms, we perform an FST. Optimal FST parameters for each individual marsquake are sought by a grid‐search on values of *V*_P_, *V*_P_/*V*s, and ray parameter that minimize correlation between the P and SV waveforms within 2/Fh and 4/Fh of the P and PP arrivals, respectively; we average the *V*_P_ and *V*_P_/*V*_S_ values when analyzing the waveforms together. Previously documented parameters obtained from the P arrivals of S0235b and S0173 events (cf., Knapmeyer‐Endrun et al., 2021) are in good agreement with our average values (Figure 4). We find that the individual FST parameters for PP arrivals are more uncertain due to the lower SNR compared to P arrivals. Therefore, we retain the average FST parameters obtained for the P arrivals and use those values for processing PPs‐RF. This is a conservative choice to prevent any bias introduced during the FST considering the observed difference in ray parameters for P and PP arrivals for our LF events is small, ranging from 0.5 to 1.1 s/° (Khan et al., 2021). We confirm that waveforms of upgoing P and SV obtained through the FST are not affected by such small ray parameter differences. Notably, differences between Ps and PPs ray parameters on Mars are much smaller than those on Earth, where the ray parameter differences can affect both time and amplitude of the mode conversions in RFs (e.g., Figure 1). Because the average Martian crust is much thicker relative to Mars' radius (Knapmeyer‐Endrun et al., 2021) and the mantle velocity gradient smaller than on the Earth, the difference in ray parameter for P and PP is much smaller on Mars, especially as epicentral distance decreases.

To compute Ps‐ and PPs‐RF, we simultaneously deconvolve the five event P waveforms from the corresponding SV waveforms to produce a single RF. To ensure the convergence in our simultaneous THBD for all five marsquake events, we monitor the evolution of the model likelihood as a function of iteration. Typically, we find that misfit starts to stabilize after ∼106 iterations as the number of Gaussian peaks combined to create the RF stops increasing and eventually converges after ∼10^7^ iterations (Figure 4). We therefore set the total number of iterations as high as 10^8^ for both Ps and PPs‐RF calculations, which is substantially larger than the number we would typically use for earthquake signals (i.e., 10^6^–10^7^ iterations) with relatively high SNR. Unfortunately, due to glitches and wind noise, we could only robustly identify Sp conversions for S0235b. Therefore, we will mainly focus on the joint analysis of Ps‐ and PPs‐RFs throughout the main text while treating the Sp‐RFs of S0235b as a separate constraint for our interpretation (see Section 4.2).

Currently, the marsquake data set suitable for RF analysis is fairly small, has low SNR, and we lack strong constraints on focal depths. Together with our limited understanding of the three‐dimensional structure of the Martian mantle and crust, this makes it difficult to uniquely associate later arriving phases with sub‐receiver structure, as opposed to other potential arrivals (e.g., depth phases or multiple branches of PP). Therefore, we choose to limit our analysis to the first 18 s of the RFs and leave analysis of the later arriving phases to future studies that can benefit from our ever improving understanding of the Martian seismic wavefield.

**Figure 5:**
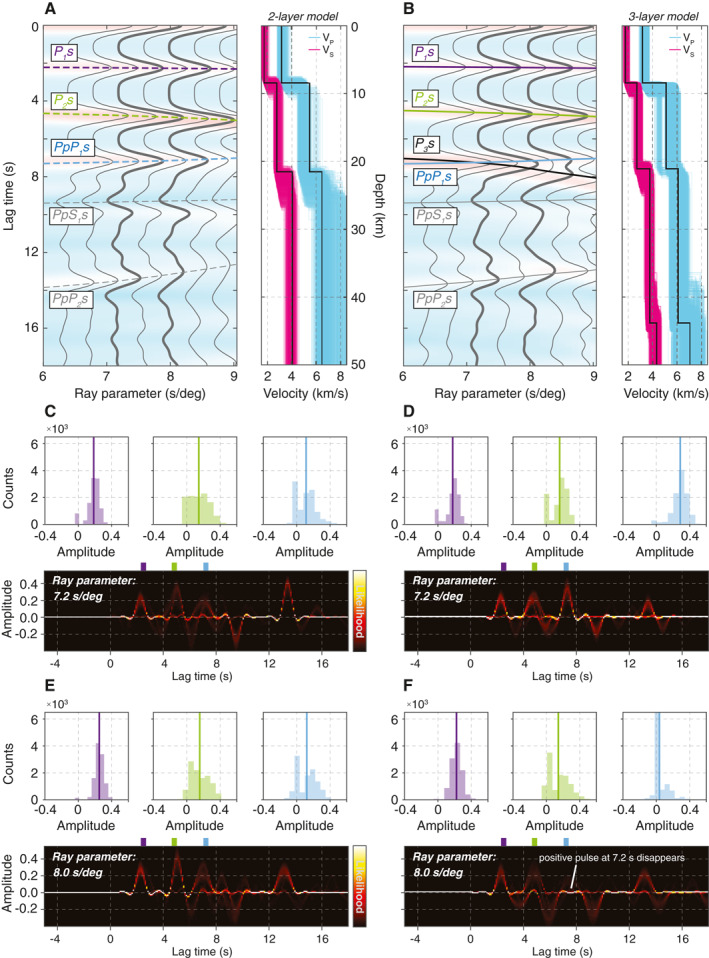
Synthetic receiver functions (RFs) using the two‐ and three‐layer Martian crustal models. Simple synthetic RF (Z/R) gather calculated based on Thomson‐Haskell matrix method using (a) two‐ and (b) three‐layer crustal models. Velocity profiles in black indicate the average of the best 5,000 models, plotted in colors, from Knapmeyer‐Endrun et al. (2021). Dashed and solid lines in panels (a and b) indicate expected arrivals based on each model, respectively. Note, the multiples (blue, PpP_1_s) exist in both models while the three‐layer model produces an additional direct conversion at the deepest interface (black, P_3_s). Traces in bold denote synthetic RFs predicted by two representative ray parameters of the incoming P‐wave from marsquakes used in this study. Comparison of the transdimensional hierarchical Bayesian deconvolution ensemble assemblages and distributions of relative amplitudes associated with observed phases at 2.4, 4.8, and 7.2 s (purple, green, and blue) based on synthetic waveforms with ray parameter of (c and d) 7.2 s/° and (e and f) 8.0 s/°. Panels on the left column correspond to the two‐layer model while those on the right correspond to the three‐layer model.

To interpret our marsquake RFs, we compute two sets of synthetic RFs: (a) Theoretical radial RF estimates based on Thomson‐Haskell matrix method (Haskell, 1962) to predict the timing of mode‐converted phases and their associated moveout as a function of ray parameter for the incoming P‐arrival (e.g., Figures 5a and 5b); and (b) Another set of synthetic RFs based on modeling marsquake waveforms with a reflectivity method (Levin & Park, 1997). Two different sets of synthetics allow us to predict the phase arrivals of the RFs from the mode conversion, and the waveform complexities in simulated waveforms as well as how they affect the corresponding THBD RFs. For our input velocity model, we use the average 1D crustal layering models inferred by previous RF analysis of S0235b, S0173a, and S0183a events and ambient noise correlations (Figures S1 and S2 in Supporting Information S1; inverted models derived from the high‐frequency RF data set described in Method D in Knapmeyer‐Endrun et al., 2021). Our synthetic waveforms are contaminated with white random noise with standard deviation of 0.01. We retain the same processing steps as for the real data to generate synthetic THBD RFs to compare to our observed Ps‐ and PPs‐RFs (e.g., Figures 5c–5h). Note however that the purpose for computing synthetic RFs is to understand the waveform behavior of the three dominant positive arrivals in our RF data given a plausible range of ray parameters for the P and PP arrivals.

Lastly, to incorporate later reverberated phases and further constrain the depth estimates of crustal interfaces, we stack the direct conversion with signals around the expected timings of the corresponding multiples (PpPs and PpSs) for ranges of crustal thickness (H), P‐wave velocity (*V*_P_), and *V*_P_/*V*_S_ (*κ*) (e.g., Cunningham and Lekić, 2019). For a given set of the *H*‐*κ*‐*V*_P_ parameters, we predict arrival times of Ps, PpPs, and PpSs relative to the direct P‐wave arrival. Subsequently, those predictions are used to correct for the time shifts on each RF solution in the THBD ensemble and the weighted sum is obtained. This triple stacking procedure (*H*‐*κ*‐*V*_P_ stack) is performed for the entire 3D parameter space across the entire RF solutions and the relative weights used for stacking follow Zhu and Kanamori (2000).

## 3 Results

### 3.1 Receiver Functions on Earth

The ensembles of Ps‐ and PPs‐RFs generated from the pair of earthquake data are shown in Figures 1b and 1c. We observe strong phases at 5.6 and 5.2 s apparent in the average Ps‐RFs (red lines, Figures 1b and 1c) of the close (Norwegian Sea, 2012) and distant event (Mariana Islands, 2016), respectively. The timing and polarity of these phases are consistent with direct Ps conversions across a Moho at a depth of 49 km, as found by EARS (Crotwell & Owens, 2005), which is substantially thicker than average continental crust (e.g., Mooney, 2015) and compatible with previous findings (e.g., Ford et al., 2016; Shen & Ritzwoller, 2016). The 0.4 s difference in lag‐time between those Ps phases is commensurate with ∼3.2 s/° difference in ray parameter due to the difference in epicentral distance. Similarly, at 6.0 s and 5.6 s, the PPs waves converted at the same boundary are delayed by 0.4 s with respect to the Ps waves due to the PP phases' larger ray parameters relative to the ray parameter for P. We chose our events so that the ray parameter of the P wave from the closer event had the same ray parameter as the PP wave from the further event (i.e., epicentral distance differing by a factor of two). As a result, the timing of the Ps conversion visible at 5.6 s lag time in our RFs from our close event coincides with that of the PPs from the more distant event. No multiple conversions from the Moho are expected within the first 15 s of our analysis window.

Figure 1d presents the theoretical trade‐off between crustal thickness (*H*) and *V*_P_/*V*_S_ (*κ*) for a given bulk crustal *V*_P_ of the region for both Ps and PPs converted phases in our RF analysis. To highlight the influence of the direct PPs conversion, we restrict our analysis to the direct conversion of P‐ and PP‐arrivals and do not include the corresponding multiples. Based on the observed difference of 3.14 s/° in ray parameters for P‐ and PP‐arrivals from our earthquakes, the corresponding *H*‐*κ* curves show negligible difference with an unresolvable crossing point between the two. The dominant frequency of teleseismic RFs is typically <1 Hz, corresponding to P wavelengths of 5–10 km, making it difficult to extract further constraints on crustal structure based on the small timing difference between Ps and PPs phases. Note that even when there is a substantial difference in ray parameter between P and PP, the timing differences remain unresolvable (see the overlap between the red, gray and blue lines in Figure 1d). The primary multiples of Ps and PPs show similarly small differences (Figure S3 in Supporting Information S1). However, because the amplitude associated with the direct conversion increases substantially with ray parameter (e.g., Figure 1c), the PPs‐RF can nevertheless provide information complementary to the Ps‐RF, especially when limited data is available (see Section 4). Stacking both Ps‐ and PPs‐RF would therefore increase the SNR and minimize potential contamination due to other scattered arrivals (Figures 1e and 1f).

### 3.2 Receiver Functions on Mars

The first RFs on Mars, calculated for marsquakes S0173a and S0235b and documented by Lognonne et al. (2020), exhibit consistent positive arrivals at lag times of 2.2–2.4 and 4.6–4.7 s. A later, positive arrival observed at ∼7 s was found to be less coherent across different realizations of the RFs (see Supporting Information of Lognonne et al., 2020). A more in‐depth RF analysis carried out by Knapmeyer‐Endrun et al. (2021) reported that the presence of the 7.2–7.5 s arrival was further supported by a third LF event, S0183a. The three consecutive positive arrivals in Ps‐RFs imply possible layering within the Martian crust, with velocity that increases with depth. However, limitations of available marsquake records and insufficient complementary geophysical constraints, hinder Knapmeyer‐Endrun et al. (2021) from distinguishing between a thin, two‐layer and a thicker, three‐layer crustal model.

Here, we incorporate three new LF marsquakes (i.e., S0407a, S0809a, and S0820a) into our RF analysis in addition to the two previously analyzed events, S0173a and S0235b. In our analysis, we benchmark the published RF results by Knapmeyer‐Endrun et al. (2021) and focus primarily on the three positive arrivals they discussed. Our Ps‐RF estimates based on the simultaneous THBD of the five LF events show stable RF features within the resulting ensemble up to 10 s (Figures 4a and 4b). Three strong positive phases are visible at 2.4, 4.8, and 7.2 s, consistent with the individual RFs of S0173a, S0235b, and S0183a (Knapmeyer‐Endrun et al., 2021). Uncertainties associated with these Ps conversions are examined at each peak and the corresponding relative amplitude to P‐wave across the entire RF solutions in the ensemble are plotted in histograms (Figure 4a). Notably, the relative amplitudes for each identified Ps phase are normally distributed with mean values of 0.26, 0.27, and 0.17 for phases at 2.4, 4.8, and 7.2 s, respectively, and standard deviations less than 0.03.

There are other arrivals also visible in our THBD RFs. We observe a negative arrival followed by a hint of small positive arrival at ∼1 s that precede the main 2.4 s arrival in the record. While these signals are stable in our THBD RFs and can be seen to become more prominent on highpass‐filtered waveforms (Figure 1; Knapmeyer‐Endrun et al., 2021), their proximity to zero lag time makes their structural interpretation sensitive to details of the FST optimization. In addition, the amplitude of the positive ∼1 s arrival is well below 1*σ* so the conversion is not robustly observed (Figure 4b). Unsurprisingly, phases arriving after 10 s display larger variability in both lag‐time and amplitude across our RF solutions in the ensemble (Figure 4a). For example, phases faintly visible at ∼11–13 and ∼16–18 s in the ensemble are not consistently seen in our RF solutions; hence we consider these features to be unreliable and do not base structural interpretations on them. On the other hand, a distinct positive peak arriving at ∼10 s appears to have stability comparable to those consistent early phases, while two negative peaks observed between 13 and 16 s show moderate variability in amplitudes.

The PPs‐RF ensemble estimates are shown in Figures 4c and 4d. Two strong phases with positive amplitude predominantly exist across our RF solutions with peaks at 2.5 and 4.8 s after the PP‐arrival. Because PP has a larger ray parameter than P for the same source, we interpret the two phases we observe in the PPs‐RFs as the same phases visible in the Ps‐RFs, with their later arrival time due only to the difference in slowness. Indeed, the apparent time delays of 0.1 and 0.2 s between phases in the Ps‐ and PPs‐RFs are similar to expectations based on the observed difference in ray parameter of 0.8 ± 0.3 s/° between the P‐ and PP‐arrivals of LF events located ∼30° away from the *InSight* lander (e.g., Khan et al., 2021). The distribution of relative amplitude measurements from the 2.5 and 4.9 s phases in our PPs‐RFs is generally broader with standard deviation being twice as large as those of the 2.4 and 4.8 s phases in Ps‐RFs. Importantly, the PPs‐RFs do not show a third positive arrival corresponding to the third positive phase visible at 7.2 s in our Ps‐RFs. Instead, in the PPs‐RFs, we do observe a later phase at ∼8.5 s with negative polarity. In contrast to the greater variability seen beyond 10 s lag time in the Ps‐RFs, the corresponding segments of the PPs‐RFs are considerably quieter particularly between 10 and 14 s, with the exception of the large negative amplitude arrival at ∼16 s.

### 3.3 Synthetic Receiver Functions

Knapmeyer‐Endrun et al. (2021) found that both two‐ and three‐layer crustal models can equally well explain the limited event data available to them. Therefore, we compute simple synthetic RFs to predict the Ps converted phases (and multiples) that would be expected based on the two plausible crustal models of Mars with different layering structures (Figures 5a and 5b). Specifically, we use crustal models derived from the high‐frequency RF datasets (Figures S1 and S2 in Supporting Information S1; Method D in Knapmeyer‐Endrun et al., 2021) to compute synthetic waveforms assuming a seismic source located 30° due east of the SEIS, because these models best predict the timings of converted phases observed in our data. In Figures 5c–5f, the computed THBD RFs based on our synthetic waveforms are shown for two representative ray parameter values of 7.2 and 8.0 s/° for P and PP‐waves, respectively.

Both the two‐ and three‐layer crustal models predict positive phases at 2.4, 4.8, and 7.2 s, as is observed in the Ps‐RFs (e.g., Figures 5c and 5d), illustrating the non‐uniqueness of structural inferences associated with the origin of the third phase. While the 2.4 and 4.8 s phases are due to the direct Ps conversion at the upper two crustal interfaces (P_1_s and P_2_s in Figures 5a and 5b), the 7.2 s phase could be a multiple of the 2.4 s phase reverberating in the shallowest layer (PpP_1_s in Figure 5a), another direct conversion from a deeper, third crustal interface (P_3_s in Figure 5b) (cf., the corresponding ray paths in Figure 5a), or result from interference of both phases. For both two‐ and three‐layer models, the observed moveouts of the P_1_s and P_2_s arrivals systematically increase as a function of ray parameter (Figures 5a and 5b). The time delay associated with these moveouts becomes significantly greater for deeper interfaces as highlighted by the P_3_s arrivals arising from the bottom interface in the three‐layer model (Figure 5b). Conversely, the opposite trend in moveout is predicted for the PpP_1_s multiples. As the result of these differences in moveout between the direct conversion and multiples, the P_3_s and PpP_1_s arrivals in the three‐layer model may be completely merged at some ray parameters, but eventually bifurcate as the ray parameter increases.

Because of this interference between the P_3_s and PpP_1_s phases, the deeper, third layer can potentially complicate the recovery of the 7.2 s phase in RFs, if the ray parameter is sufficiently large. Therefore, we investigate the stability of THBD RFs computed from synthetic waveforms, for limiting ray parameter values of the incoming P‐waves of 7.2 and 8.0 s/° (e.g., bold traces in Figures 5a and 5b). For each ray parameter, we compute a series of ensemble THBD RFs as we vary the length of our analysis window on the P‐component data between 3 and 20 s and explore the variability across the entire RF solutions (e.g., Figures 5c–5f). We find synthetic RF computed for both two‐ and three‐layer models to be consistent with each other when the ray parameter of the incoming P wave is equivalent to 7.2 s/°, the average ray parameter estimated by the FST for the five marsquakes (Figures 5c and 5d). The three phases at 2.4, 4.8, and 7.2 s are generally stable throughout different ensembles and insensitive to the window length used in our analysis. Note that in the two‐layer model, the complexity of the 7.2 s phase in the synthetic RFs is due to near‐critical reflection of the PpP_1_s phase, and not to interference with any other phase. When the ray parameter is 8.0 s/°, the two‐ and three‐layer models predict incompatible RF waveforms (Figures 5e and 5f). None of the RFs obtained from the three‐layer model synthetics contained all three expected phases converting at the model interfaces. When the ray parameter approaches 8.0 s/°, the 7.2 s phase disappears in nearly all of the solutions in the RF ensemble, affected by the divergence between P_3_s and PpP_1_s arrivals (Figures 5g and 5h). Though the accurate timing and interaction related to those arrivals may vary considerably as velocity and thickness changes, this synthetic test highlights that the stability of the resulting RF for the three‐layer model is strongly dependent on waveform complexities associated with the crossover of P_3_s and PpP_1_s arrivals. This implies that examining how RF waveforms change with ray parameter near the threshold value of 8.0 s/° can provide strong constraints on intra‐crustal structure.

In the aforedescribed analysis, we used mantle velocities that are somewhat lower than those recently published by Khan et al. (2021) (Figure S4 in Supporting Information S1). In the three‐layer case, the higher mantle *V*_P_ would imply that the P or PP waves with a ray parameter greater than ∼7.5 s/° would not enter the mantle and no conversion across the bottom interface would be predicted. In that case, we would expect to see a positive PpP_1_s phase at 7.2 s, which would no longer be masked by interference with the non‐existent P_3_s phase. Indeed, even in a two‐layer model, the PpP_1_s phase should be seen in the RFs near 7.2 s. If the ray parameter of PP waves analyzed here is greater than mantle slowness in the Khan et al. (2021), then we cannot explain the absence of a positive ∼7.2 arrival in the PPs‐RFs.

There are other phases apparent in our synthetics arriving after the three positive peaks discussed above. These include a negative phase at ∼9.5 s that corresponds to PpS_1_s from the first interface and a positive arrival at ∼13.5 s which is PpP_2_s from the second interface (Figures 5a and 5b). All other multiples arrive outside our analysis window. These are also visible in the THBD synthetic RF counterparts (Figures 5c–5f).

## 4 Discussion and Interpretation

### 4.1 Constraints From Multiples

Our THBD Ps‐RFs using five LF events consistently show three positive phases, the first two of which are unequivocally interpreted as the direct conversions across two intra‐crustal interfaces (P_1_s and P_2_s; Lognonne et al., 2020; Knapmeyer‐Endrun et al., 2021). Weaker, but less stable phases with opposite polarities are also evident at later lag times (>10 s) suggesting potential multiples, such as PpPs and PpSs, generated across the same interfaces. To further constrain the depth estimates of crustal interfaces, we incorporate those later reverberated phases and conduct *H*‐*κ*‐*V*_P_ analysis for the range of layer thicknesses and *V*_P_ in the model ensembles of Knaymeyer‐Endrun et al. (2021) (e.g., Figures 5a and 5b; inverted models derived from the high‐frequency RF data set described in Method D in Knapmeyer‐Endrun et al., 2021).

**Figure 6:**
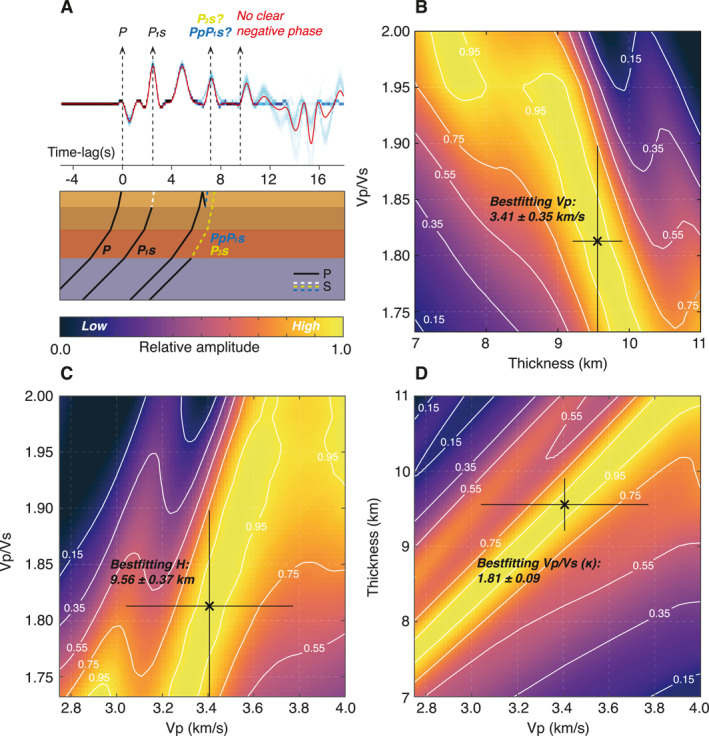
*H*‐*κ*‐*V*_P_ triple stacking of the P‐to‐S (Ps)‐receiver functions (RFs) focused on the first interface. (a) Ensemble Ps‐RF results from Figure 4b with timings of the phases related to the direct and multiples associated with the uppermost crustal interface corresponding to the best‐fitting parameters resulting from the *H*‐*κ*‐*V*_P_ analysis in panels (b–d). See Figure S7 in Supporting Information S1 for the enlarged version of this panel including labels of all of the converted phases discussed in the main text. Colorbar shown beneath panel (a) denotes relative amplitude of the *H*‐*κ*‐*V*_P_ in panels (b–d). Schematic raypaths of the analyzed phases are shown below. As discussed in the main text, the origin of the 7.2 s phase is debated. Note, the presence of the negative phase associated with PpS_1_s multiple is unclear. (b–d) The cross‐sections sliced through the parameter space at the maximum of the *H*‐*κ*‐*V*_P_ triple stack. Black cross with cross‐hair denotes the maximum value ±1*σ*.

Because multiples from our PPs‐RFs were not readily identifiable, here, we primarily focus our analysis on the Ps‐RF ensemble. If we assume that the 7.2 s phase of the Ps‐RF data is the PpP_1_s multiple of P_1_s reverberated within the uppermost crustal layer (PpP_1_s), our best‐fitting *H*‐*κ*‐*V*_P_ parameters for this layer are found at *V*_P_ = 3.41 ± 0.35 km/s, *H* = 9.56 ± 0.37 km, and *κ* = 1.81 ± 0.09 (Figure 6), which is in general agreement with either two‐ or three‐layer model solutions from the inversion (Knapmeyer‐Endrun et al., 2021). We do not observe the PpS_1_s multiple in our data (rightmost arrow in Figure 6a), which may be due to interference with the ∼10 s positive arrival.

We highlight that the positive polarity of the ∼10 s phase is necessary in our RF solutions by examining the SV waveform predictions from the opposite polarity (Figure S5 in Supporting Information S1); a negative polarity ∼10 s phase fits the data less well. Meanwhile, it is tempting to relate the stable ∼10 s positive phase and a negative phase between 13 and 16 s to be the PpP_2_s and PpS_2_s of the 4.8 s phase from the second interface (Figure S6a in Supporting Information S1). If these two multiples with the expected polarity reversal originate at the second interface, the depth to this second layer implied by the triple stack would be 25.5 ± 0.8 km (Figure S6 in Supporting Information S1), which is generally consistent with the depth range suggested by previous studies using LF marsquakes (Knapmeyer‐Endrun et al., 2021; Lognonne et al., 2020). However, while the depth estimate is also compatible with those constrained by autocorrelation functions of ambient noise and the high frequency family marsquakes (Compaire et al., 2021; Schimmel et al., 2021), the best‐fitting *V*_P_ is unreasonably high for crustal material on Mars (Figure S6 in Supporting Information S1). Therefore, the ∼10 s phase is most likely not a multiple of the second layer, and may contaminate the *H*‐*κ*‐*V*_P_ stack shown in Figure S6 in Supporting Information S1. Alternatively, the ∼10 s phase may be generated by the direct conversion due to a deep (∼60–100 km) interface of unknown origin within the Martian mantle. Unfortunately, similar *H*‐*κ*‐*V*_P_ analysis with P_2_s and P_3_s phase using their corresponding multiples beyond 10 s is not feasible due to large variability of our THBD solutions, when the expected multiples would arrive.

### 4.2 Evidence for a Three‐Layer Martian Crust

Our Ps and PPs RF results differ most dramatically near 7.2 s lag time; a strong positive signal is present in the Ps‐RFs but this signal disappears in the PPs‐RF counterparts (Figures 3d and 4b). Such instability of the 7.2 s phase is similarly observed in synthetic RFs only for the three‐layer model and only when the ray parameter of the incoming P waves reaches 8.0 s/° (Figure 5f). The ray parameter of the P waves for the marsquakes analyzed here are expected to be in the 7.0–7.4 s/° range (Lognonne et al., 2020), just below this threshold ray parameter value at which expected arrivals of P_3_s start to diverge from the PpP_1_s multiples, producing differences in RF appearance. As documented by Khan et al. (2021), the ray parameters for PP‐waves are observed to be 0.8 ± 0.3 s/° higher than for P‐waves generated by marsquakes 30° away from the *InSight* lander, which is about an order of magnitude smaller than the corresponding PP—P ray parameter difference on Earth. Therefore, the absence of the 7.2 s phase from our PPs‐RFs may indicate that our PPs‐ and Ps‐RFs bracket this threshold ray parameter value, and are, somewhat counterintuitively, evidence for the presence of the third interface.

**Figure 7:**
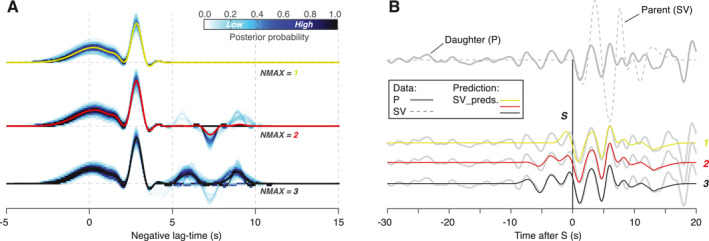
Transdimensional hierarchical Bayesian deconvolution (THBD) S‐to‐P (Sp)‐receiver functions (RFs) analysis of the S0235b marsquake. (a) A series of ensemble Sp‐RFs combining all models of individual low‐frequency event S0235b when the maximum number of Gaussian pulses (i.e., maximum number of pulses) allowed in THBD is set as 1, 2, and 3 (the average RF for each case is color coded by green, red, and black). (b) Comparison of the S0235b P‐component data and the corresponding waveform predictions generated by the average RFs in panel (a). Bold and dashed gray lines indicate P‐ and SV‐component raw waveforms, respectively. Note, only the average RF with three positive phases (black, a) allows the prediction that fits all three S‐precursors (see those records prior to the S‐arrival denoted by black dashed line) in the raw data. Chi‐squared misfit between the Sp‐RFs and the corresponding predictions from RFs with different maximum number of pulses values does not decrease as additional pulses past the third are allowed (see Figure S8 in Supporting Information S1).

Previously reported Sp‐RFs calculated for S0235b provide another piece of evidence to verify the necessity of the third interface. The THBD Sp‐RFs of S0235b in Knapmeyer‐Endrun et al. (2021) shows three positive phases similar to those observed in our Ps‐RFs. We limit the maximum number of pulses (NMAX) allowed in the THBD and compute three ensemble Sp‐RFs of S0235b, where NMAX is set to 1, 2, or 3, and plot the resulting RF ensembles in Figure 7a (flipped in time for easier comparison to Ps‐RFs). We then compare the P waveforms predicted by convolving the RFs with the SV waveform (Figure 7b) with the observed P‐component signal. We see that the two additional positive phases present in the NMAX = 3 RFs are required to fit the precursory P‐component waveform prior to the main S arrival. Chi‐squared misfit between the Sp waveform and the corresponding predictions from Sp‐RFs with different NMAX values does not decrease as additional pulses past the third are allowed (Figure S8 in Supporting Information S1). If the earliest S‐precursor is due to multiples of any direct Sp phases, such a hypothetical signal would arrive after the main S phase observed in the raw data. Therefore, the required third positive phase present in the Sp‐RFs implies the existence of a third crustal interface, and thus supports the three‐layer model for the crust beneath the *InSight* lander.

Importantly, the seismic evidence for a three‐layer crust on Mars is supported by other lines of evidence, as discussed in Knapmeyer‐Endrun et al. (2021). First, the greater average crustal thickness implied by the three‐layer model is compatible with the estimated amount of heat producing elements within the Martian crust as constrained by gamma ray spectroscopy observations (the two‐layer model is less consistent) (e.g., Hahn et al., 2011; Taylor, 2013) and thermal evolution modeling. Second, the implied crustal densities obtained from global crustal thickness modeling using the three‐layer model are consistent with the known compositions of Martian basalts when including a small amount of porosity (e.g., Baratoux et al., 2014). Without the presence of this additional layer, both a substantial enrichment in crustal heat producing elements and a lower bulk crustal density (<2,850 kg m^−3^) would be required for the Martian crust (Knapmeyer‐Endrun et al., 2021). While such a scenario is not impossible, it requires a substantial reservoir of enriched feldspathic and/or silicic rocks in the Martian crust that have not yet been conclusively identified.

In our preferred three‐layer model of the Martian crust, the deepest interface represents the crust‐mantle interface (i.e., the Moho) beneath the *InSight* landing site. The nature of the other two shallower interfaces is less certain. One of the interfaces could potentially represent a transition between fractured and unfractured materials. As shown by Gyalay et al. (2020), viscous flow of crustal materials could have removed any pre‐existing porosity deep in the crust when the heat flow of Mars was higher in the past. As a result of the exponential dependence of viscosity on temperature, the transition between porous and non‐porous materials is expected to be sharp (about a kilometer). Though Gyalay et al. (2020) argued that the shallowest 10 km discontinuity could represent this transition, the mechanism could alternatively explain the intermediate interface instead (though with a somewhat lower heat flow). The other, remaining interface could potentially represent a lithological transition between a pre‐existing crust and overlying near surface materials such as lava flows and sediments. If this is the origin of the shallowest interface, then this would imply the deposition of about 10 km of materials in the northern lowlands after the crust in this region formed.

### 4.3 Implication for Using PPs Receiver Functions

Fundamental limitations to the utility of RF analysis for structural inferences may include: (a) limited seismic source availability, (b) sparse station coverage in planetary exploration, and (c) idiosyncrasies and associated complexities imbedded in planetary seismic data (e.g., Kim et al., 2021). In terrestrial seismology, similar challenges can be partially alleviated by stacking multiple measurements from a number of earthquakes, employing array‐based methods, or even deploying additional seismic stations to (temporarily) improve data coverage. No such measures are available to us in the context of RF analysis for Mars. In this paper, we show two ways of increasing confidence in structural interpretations based on RF analysis. First, our THBD based approach for computing RFs systematically quantifies uncertainties of each feature within the RFs, and estimates the parameters describing the background noise in the raw data. Second, by detecting and analyzing PPs conversions, we are able to extract information complementary to that garnered from first arriving P‐ or S‐waves. Given the limited number of high‐quality marsquakes recorded by *InSight*, the use of PPs‐RFs allows us to maximize the utility of the existing sparse data set and provide new constraints on the crustal structure of Mars.

**Figure 8:**
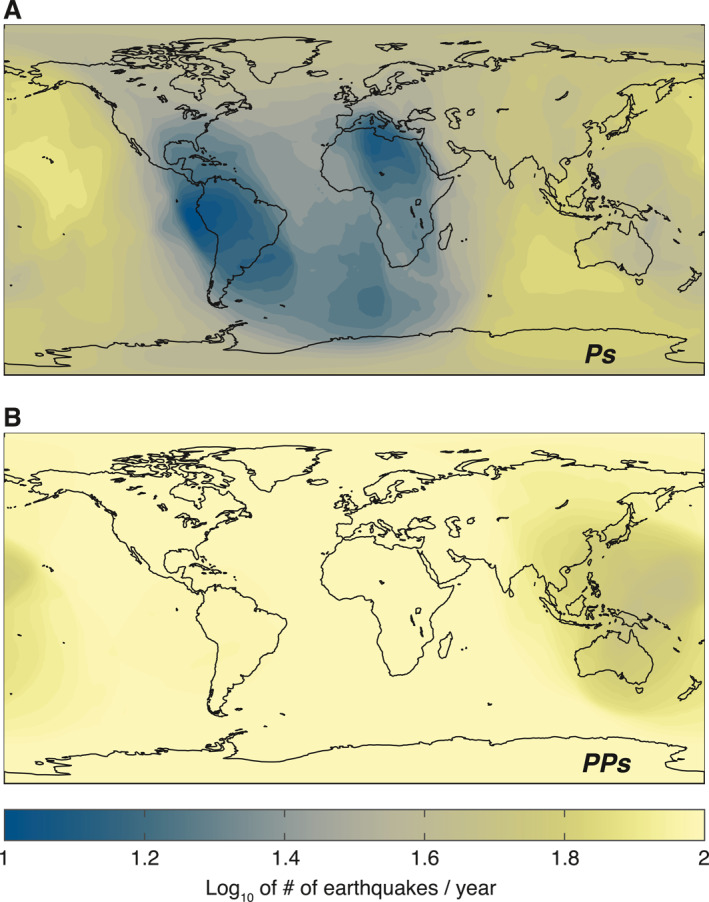
Yearly expected number of earthquake appropriate for (a) P‐to‐S (Ps) and (b) P‐waves (PPs) analysis on Earth. Given the distribution of sufficiently large (Mw > 6) and shallow (depth < 300 km) earthquakes, the number of events suitable for computing Ps‐ and PPs‐RFs varies geographically due to the difference in epicentral distance ranges of 35–85 and >50° appropriate for crustal Ps and PPs analysis, respectively. The maps show complementary coverage with more events suitable for PPs analysis being expected in locations where fewer events are appropriate for Ps analysis.

Predominantly, RF analysis on Earth is performed with Ps‐ and Sp‐RFs derived from the direct P‐ or S‐waves from large (MW > 6) teleseismic events. Based on annual rate and geographic distribution of earthquakes, we compute coverage maps (e.g., Kohler et al., 2020) showing the number of earthquakes appropriate for RF analysis of the crustal structures on Earth (Figure 8). Outside the Atlantic basin and the surrounding continental regions including both South America and Africa, seismometers will record high numbers of earthquakes suitable for computing Ps‐RFs (Figure 8a). Because PP phases can be observed across a greater range of epicentral distances, a larger number of earthquakes can be used to compute PPs‐RFs, resulting in more uniform global coverage (Figure 8b), compared to Ps. Despite their larger Ps conversion coefficients, PPs‐RFs are more challenging to compute compared to Ps‐RFs, due to their more complicated and weaker parent waveforms. Nevertheless, the improvements in coverage can enhance constraints on planetary interior structure even for Earth, especially in regions where relatively limited observations are available.

## 5 Conclusions

In this study, we explore the utility of the PPs‐RFs using *InSight* seismic recordings on Mars. We first demonstrate the reliability of the PPs‐RFs using earthquake data. Our analysis with terrestrial data illustrates how the PPs‐RFs can be useful and provide complementary information when only sparse data is available to constrain crustal structure. To obtain robust Ps‐ and PPs‐RFs on Mars, we take five high‐quality LF marsquakes and carry out THBD on these events simultaneously. Robust RF characteristics in the Ps‐RF ensemble are clear positive phases at 2.4, 4.8, and 7.2 s relative to the P‐arrivals, verifying the previous RF analysis based on the events acquired earlier in the mission. The later phases at ∼10 and 13–16 s observed in our Ps‐RF data show comparable stability to those three earlier phases and exhibit the polarity reversal that would be expected if they represent primary multiples of the 4.8 s phases. However, because its timing requires an unrealistically high *V*_P_ from our *H*‐*κ*‐*V*_P_ analysis, it is unlikely that the ∼10 s phase is a multiple of the second layer. Instead, one could interpret this stable phase as the direct conversion across a deeper interface within the Martian mantle whose origin is unknown and warrants further study. While counterparts to both the 2.4 and 4.8 s phases are apparent in PPs‐RF, the absence of 7.2 s phase in our PPs‐RF data together with the Sp‐RF of S0235b provides evidence for a three‐layer crustal structure beneath the *InSight* lander on Mars. While the deepest interface of the three‐layer crust represents the crust‐mantle interface, the other two interfaces at shallower depths could represent a sharp transition between either fractured and unfractured materials or thick basaltic flows and pre‐existing crustal materials.

## Supporting information

Supporting Information S1

## Data Availability

The *InSight* seismic waveform data are available from the IPGP Datacenter, IRIS‐DMC (InSight Mars SEIS Data Service, 2019; http://www.iris.edu/hq/sis/insight) and the NASA PDS (https://pds-geosciences.wustl.edu/missions/insight/). The data are produced and visualized with Python and MATLAB programming languages.
