# Supplementary material for: Improving Constraints on Planetary Interiors With PPs Receiver Functions
Source: J Geophys Res Planets. 2021 Nov 2;126(11):e2021JE006983. doi: 10.1029/2021JE006983 (PMC8597591; doi:10.1029/2021JE006983)
Supplement: Supplementary file 1 — Supporting Information S1 [file JGRE-126-e2021JE006983-s001.pdf]

JGR: Planets

Supporting Information for

Improving constraints on planetary interiors with PPs receiver functions

D. Kim[1], V. Lekić[1], J.C.E. Irving[2], N. Schmerr[1], B. Knapmeyer-Endrun[3], R. Joshi[4], M. Panning[5], B. Tauzin[6,7], F. Karakostas[1,8], R. Maguire[1,9], Q. Huang[1,10], S. Ceylan[11], A. Khan[11], D. Giardini[11], M. A. Wieczorek[12], P. Lognonné[13], and W. B. Banerdt[5]

[1]Department of Geology, University of Maryland, College Park, MD 20742, USA; [2]School of Earth Sciences, University of Bristol, Bristol, UK; [3]Bensberg Observatory, University of Cologne, Bergisch Gladbach, Germany; [4]Max Planck Institute for Solar System Research, Justus-von-Liebig-Weg 3, 37077 Göttingen, Germany; [5]Jet Propulsion Laboratory, California Institute of Technology, Pasadena, CA, USA; [6]Université de Lyon, Laboratoire de Géologie de Lyon, UCBL, ENSL, CNRS, LGL-TPE, Villeurbanne, France; [7]Australian National University, Research School of Earth Sciences, Acton, ACT 22601, Australia; [8]Istituto Nazionale di Geofisica e Vulcanologia, Sezione di Bologna, Bologna, Italy; [9]Department of Computational Mathematics, Science, and Engineering, Michigan State University, East Lansing, MI, USA; [10]Department of Physics, New Mexico State University, Las Cruces, New Mexico, USA; [11]Institute of Geophysics, ETH Zürich, Zürich, Switzerland; [12]Université Côte d'Azur, Observatoire de la Côte d'Azur, CNRS, Laboratoire Lagrange, France; [13]Université de Paris, Institut de Physique du Globe de Paris, CNRS, Paris, France

Contents of this file

Figures S1 to S8

Introduction

The supporting information below includes:

- The 2- and 3-layer crustal model ensembles discussed in the main text as well as the high-frequency HF data (Knapmeyer-Endrun et al., 2021) used in the inversion and its corresponding waveform prediction (Fig. S1-2)
- Trade-offs between thickness (H) and V_P/V_S (k) for Ps and PPs analysis on Earth for the bulk crustal V_P of 6.5 km/s (Fig. S3)
- The first 100 km of Mars's upper mantle velocity model ensembles in Khan et al. (2021) and the distribution of V_P and V_S values just below the crust-mantle boundary (Fig. S4)
- Waveform prediction analysis of the ~10 s phase observed in Fig. 3B (Fig. S5)
- H-κ-V_P triple stacking of the Ps-RFs focused on the second interface (Fig. S6)
- Ensemble THBD Ps-RFs of marsquakes with the associated raypaths discussed in the main text (Fig. S7)
- Misfit of Sp waveforms as a function of maximum number of Gaussian pulses in THBD RF anlaysis (Fig. S8)

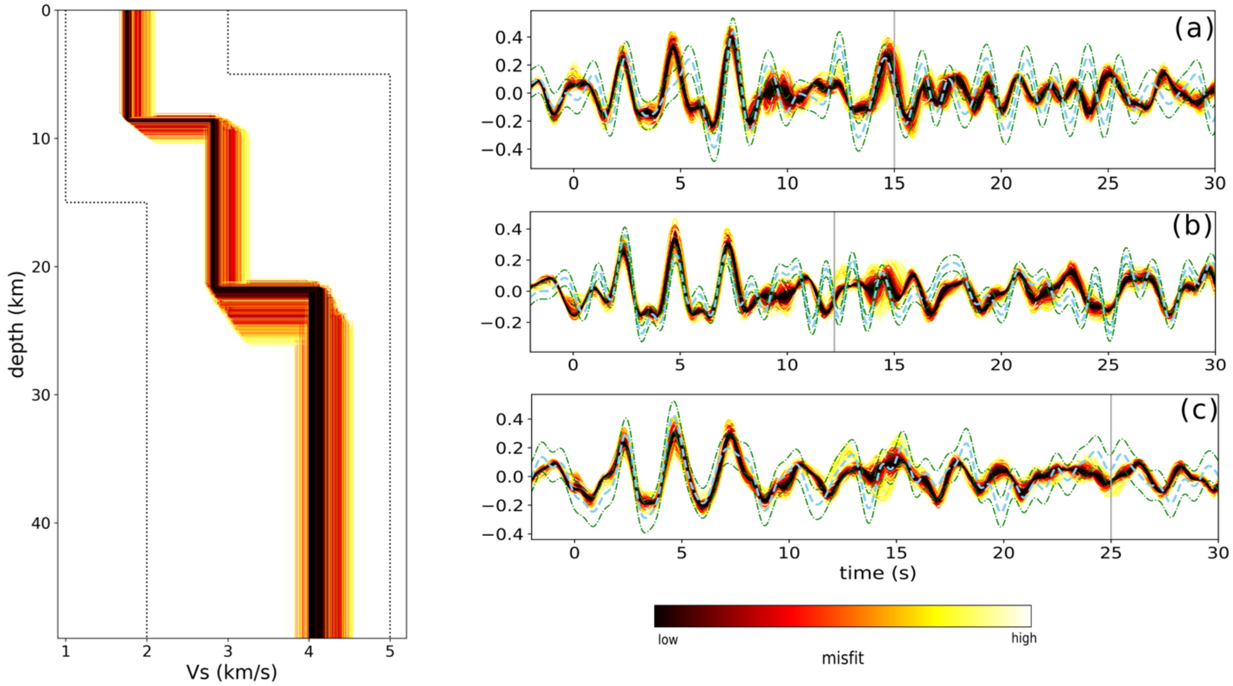

Figure S1. Mars's 2-layer model ensembles from the high-frequency RF dataset. Left: 1D velocity profiles showing V_S as a function of depth for 2 constant-velocity layers over a half-space. The dotted lines represent the limits of the parameter space. Right: Modeled RF waveforms for ray parameter (a) 6.6 s/deg, (b) 7.05 s/deg, (c) 7.15 s/deg. The blue dashed curve represents the observed RF waveform and the solid green curves show the uncertainty in observation computed from pre-event noise. Thin grey vertical lines denote the end of the misfit window. See Knapmeyer-Endrun et al. (2021) and Joshi et al (2021) for details on RF inversion.

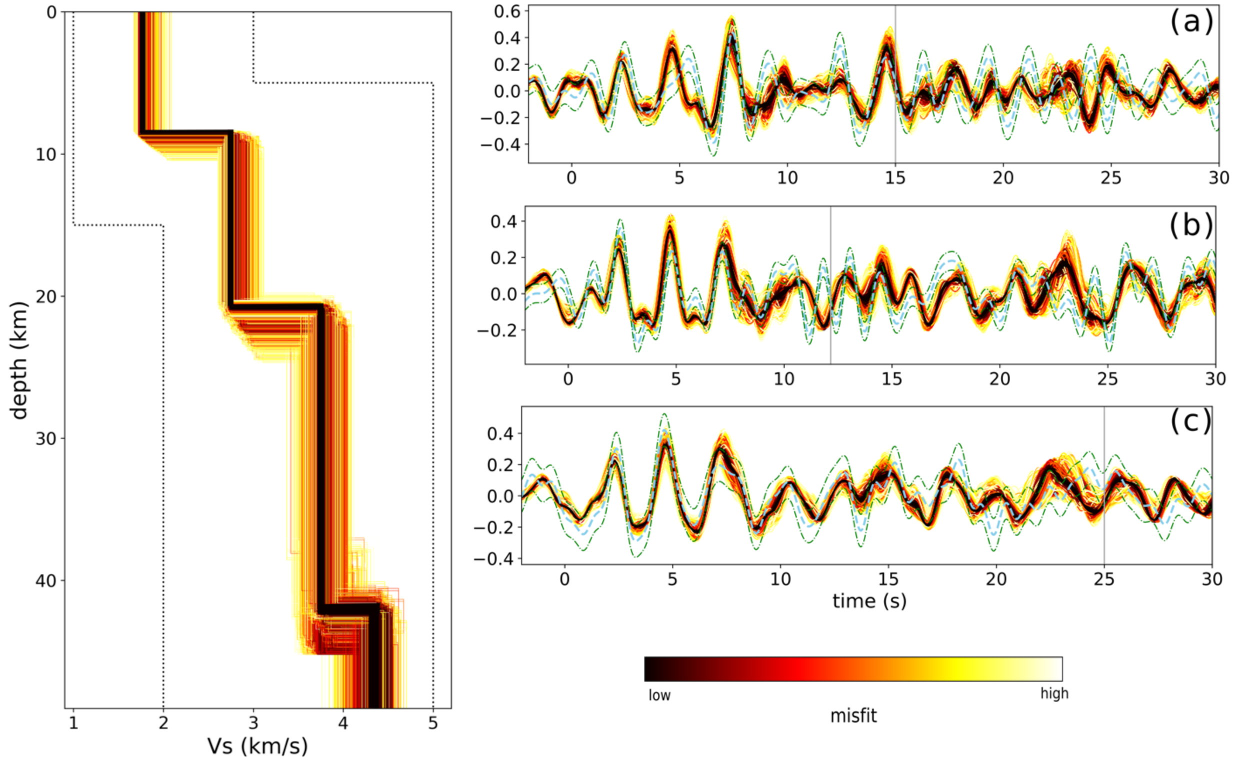

Figure S2. Mars's 3-layer model ensembles from the high-frequency RF dataset. Details as Fig. S1 for 3 constant-velocity layers over a half-space.

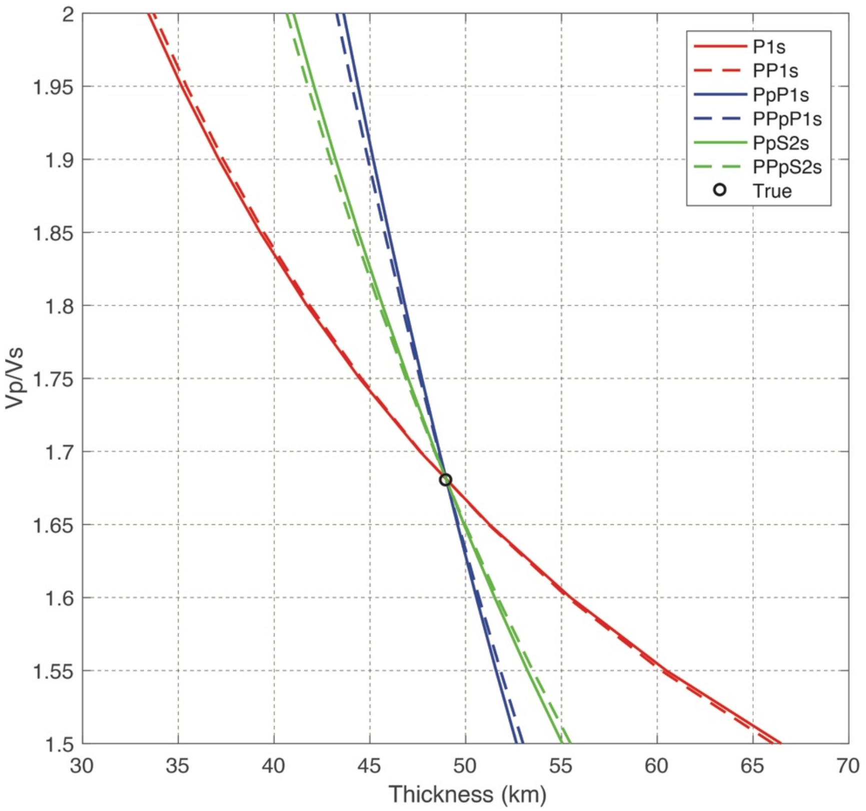

Figure S3. Trade-offs between thickness (H) and V_P/V_S (k) for Ps and PPs analysis. Theoretical H-κ curves for Ps (solid) and PPs (dashed) phases and their corresponding primary multiples for the bulk crustal V_P of 6.5 km/s. See section 3.1 Receiver functions on Earth for details.

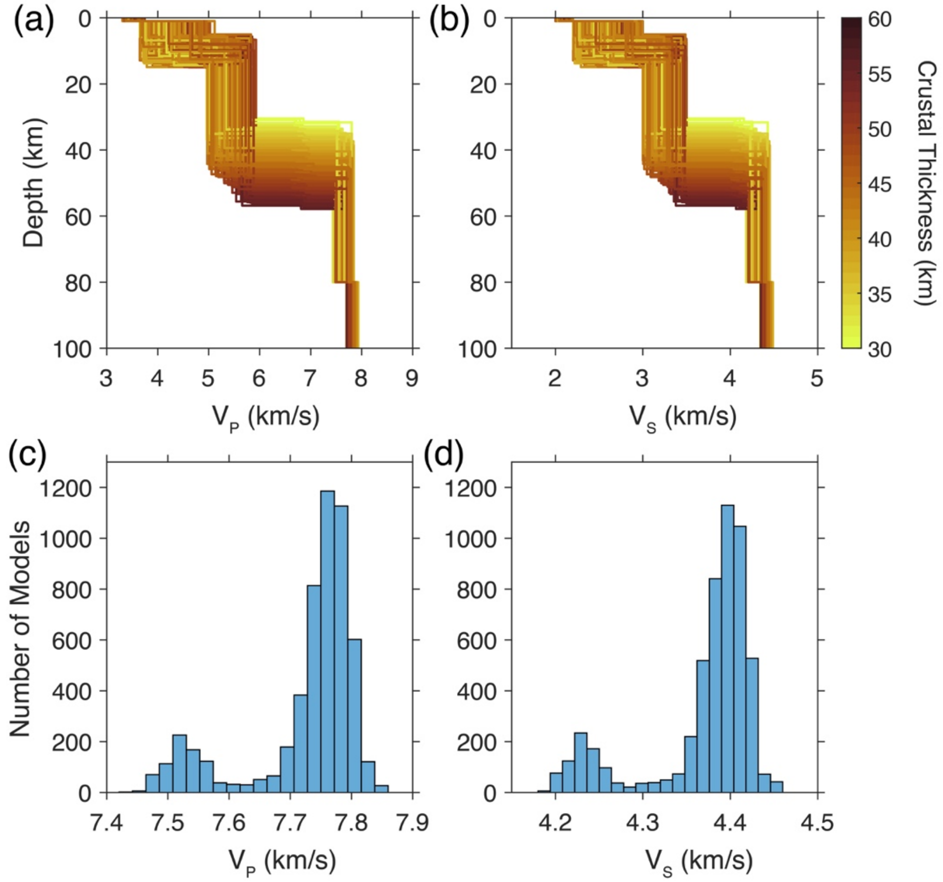

Figure S4. The first 100 km of Mars's upper mantle model ensembles in Khan et al. (2021). (A-B) Inverted P- and S-wave velocity profiles using both geophysical and seismic parameterization. See Khan et al. (2021) for details on travel time identification and measurements, prior information, parameterization and inversion methods. (C-D) Histograms of the V_P and V_S values for all models in (A-B) at depths below the crust-mantle boundary.

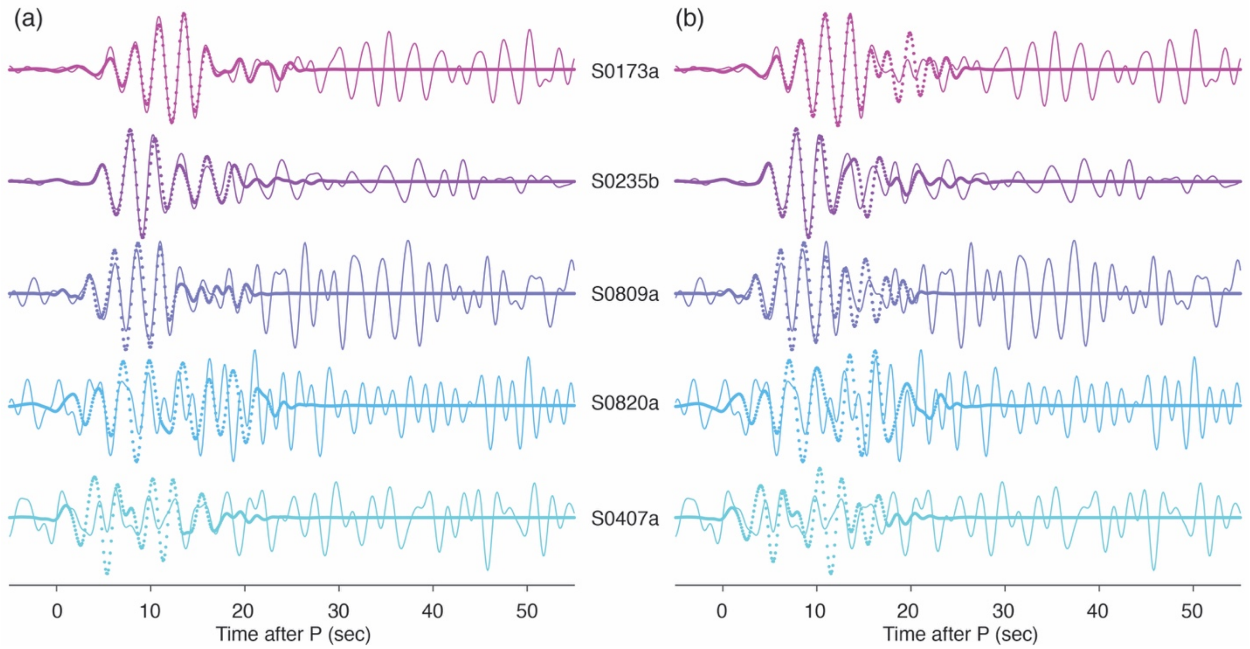

Figure S5. Waveform prediction analysis of the ~10s phase in Ps-RF. (A) Comparison of the SV-component event waveforms of the five marsquakes (solid lines) and the corresponding waveform predictions (dotted line) generated by the average, truncated Ps-RF up to 11 s. (B) Same as (A) but prediction results are computed with hypothetical negative polarity for the ~10s phase.

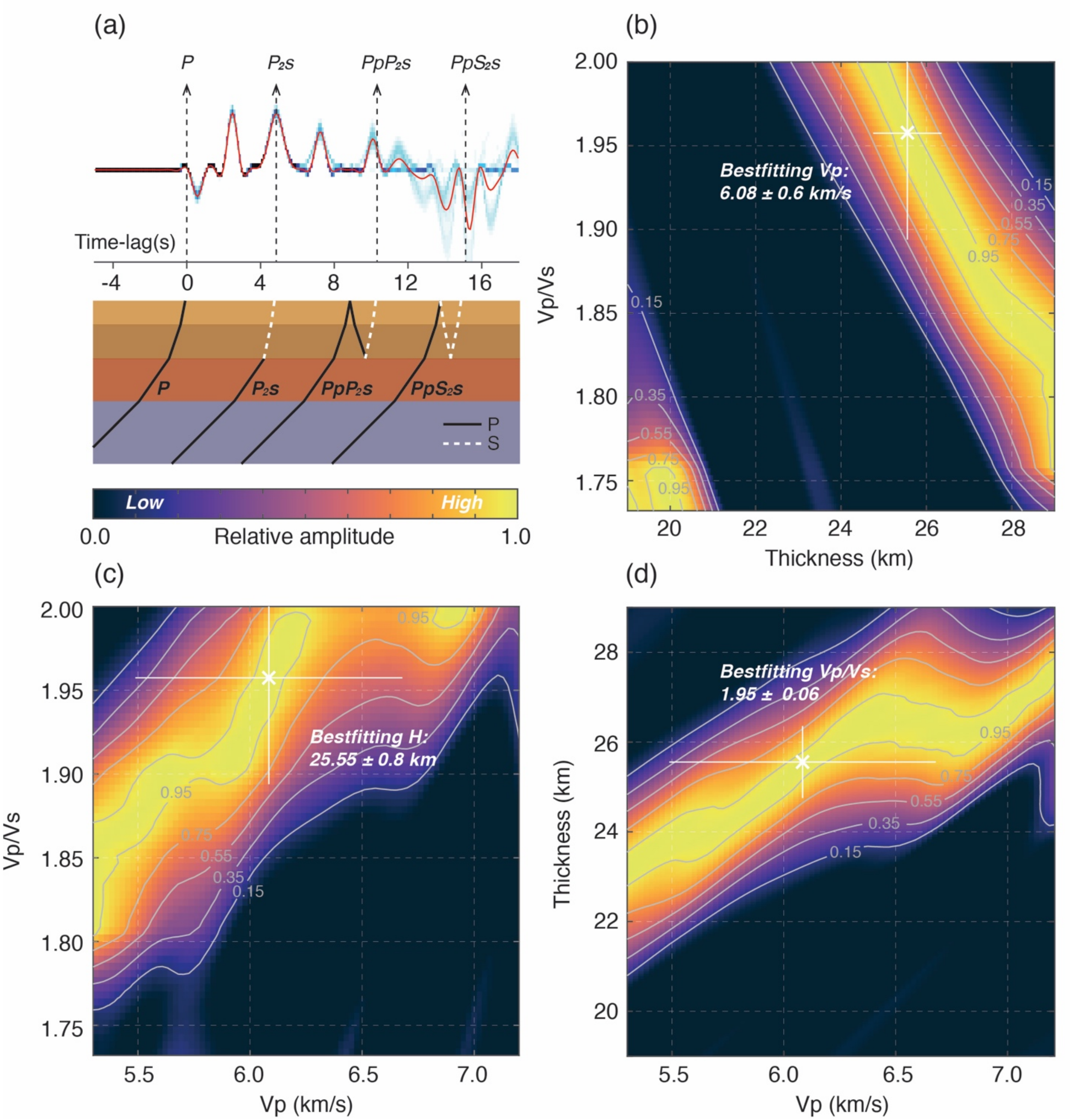

Figure S6. H-κ-V_P triple stacking of the Ps-RFs focused on the second interface. Same as Fig. 5 but results on the H-κ-V_P analysis here are focused on the second interface as we treat it as a single layer from the surface. (A) Timings of the phases related to the direct and multiples associated with the second crustal interface corresponding to the best-fitting parameters (which represent the root-mean-square V_P and κ accounting for the whole crustal thickness of H ranging from the surface to the second interface) and resulting from the H-κ-V_P analysis in (B-D). Schematic raypaths of the analyzed phases are shown below. (B-D) The cross-sections sliced through the parameter space at the maximum of the H-κ-V_P triple stack. White cross with cross-hair denotes the maximum value ± 1σ.

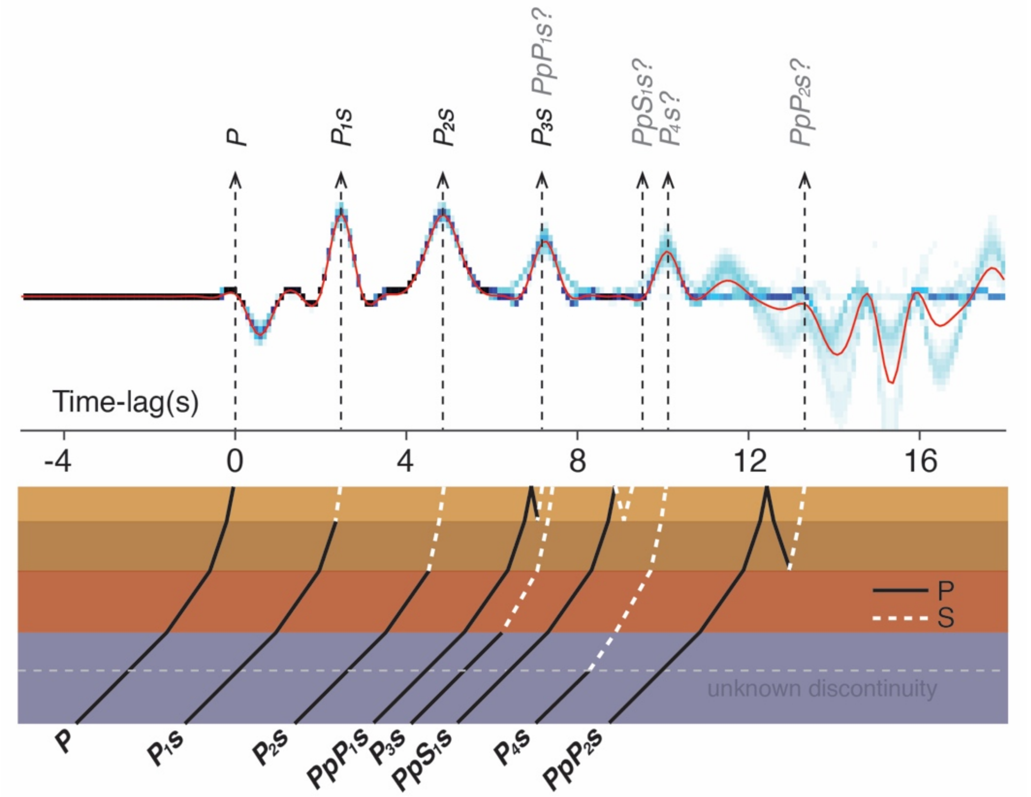

Figure S7. Ensemble THBD Ps-RFs of marsquakes with the associated raypaths. Ensemble Ps-RF results from Fig. 4B with timings of the phases related to the direct conversions and their primary multiples. All other multiples not shown in the figure arrive outside the analysis window. Robustness of identification of the phases labeled in gray is discussed in the main text.

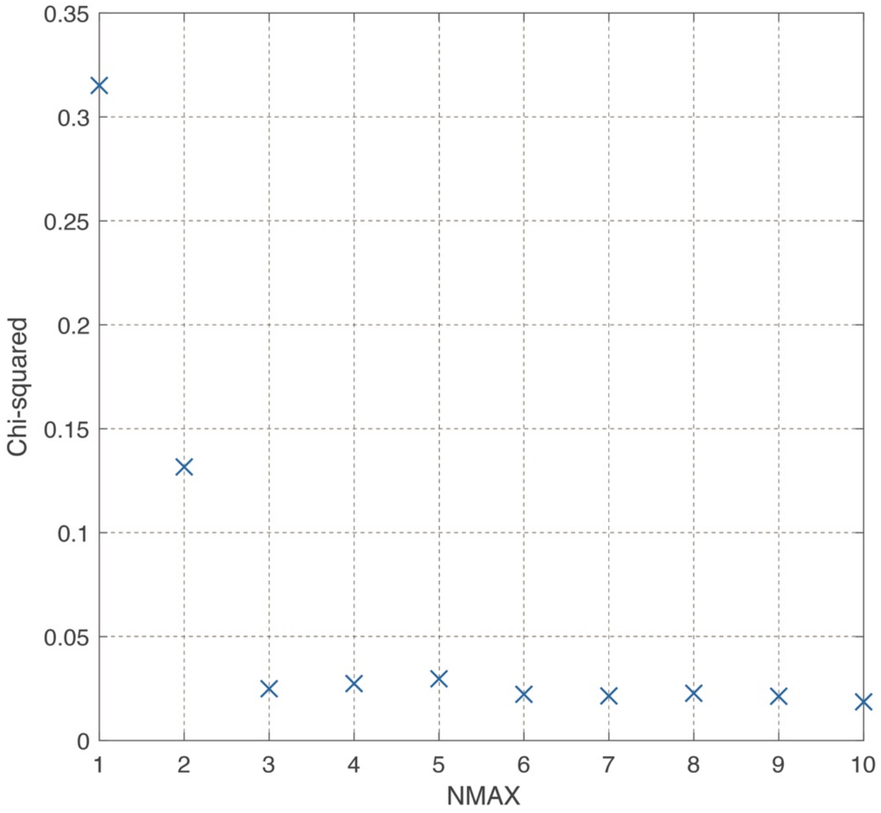

Figure S8. Misfit of Sp waveforms as a function of maximum number of Gaussian pulses. Chi-squared misfit between the Sp waveform and the corresponding predictions from Sp-RFs with different maximum number of Gaussian pulses (i.e., NMAX) does not decrease as additional pulses past the third are allowed. See section 4.2 Evidence for a 3-layered Martian crust for details.
